# Embedded Complexity of Evolutionary Sequences

**DOI:** 10.3390/e26060458

**Published:** 2024-05-28

**Authors:** Jonathan D. Phillips

**Affiliations:** Earth Surface Systems Program, University of Kentucky, Lexington, KY 40506, USA; jdp@uky.edu; Tel.: +1-859-285-2222

**Keywords:** historical sequence, evolutionary sequence, embedded complexity, spectral radius, state-and-transition model, algebraic graph theory

## Abstract

Multiple pathways and outcomes are common in evolutionary sequences for biological and other environmental systems due to nonlinear complexity, historical contingency, and disturbances. From any starting point, multiple evolutionary pathways are possible. From an endpoint or observed state, multiple possibilities exist for the sequence of events that created it. However, for any observed historical sequence—e.g., ecological or soil chronosequences, stratigraphic records, or lineages—only one historical sequence actually occurred. Here, a measure of the embedded complexity of historical sequences based on algebraic graph theory is introduced. Sequences are represented as system states *S*(*t*), such that *S*(*t* − 1) ≠ *S*(*t*) ≠ *S*(*t* + 1). Each sequence of *N* states contains nested subgraph sequences of length 2, 3, …, *N* − 1. The embedded complexity index (which can also be interpreted in terms of embedded information) compares the complexity (based on the spectral radius *λ*_1_) of the entire sequence to the cumulative complexity of the constituent subsequences. The spectral radius is closely linked to graph entropy, so the index also reflects information in the sequence. The analysis is also applied to ecological state-and-transition models (STM), which represent observed transitions, along with information on their causes or triggers. As historical sequences are lengthened (by the passage of time and additional transitions or by improved resolutions or new observations of historical changes), the overall complexity asymptotically approaches *λ*_1_ = 2, while the embedded complexity increases as *N*^2.6^. Four case studies are presented, representing coastal benthic community shifts determined from biostratigraphy, ecological succession on glacial forelands, vegetation community changes in longleaf pine woodlands, and habitat changes in a delta.

## 1. Introduction

Ecosystems evolve. They change over time in a systematic, non-random, but also non-deterministic, way and are affected by various types of selection on a broad range of scales, as well as by complex nonlinear dynamics, chance events such as disturbances and mutations, and (at all but the shortest time scales) changing boundary conditions. Ecosystem evolution is path-dependent and historically and geographically contingent. In terms of predicting ecosystem development from a specified starting point, multiple pathways and outcomes are possible (e.g., [1,2,3,4,5]).

With respect to explaining or interpreting an ecosystem at a given time or place, there may be many potential starting points and multiple developmental trajectories, historical sequences, or other forms of evolutionary pathways that could have led to the observed system state. However, there is only one that produced the observed ecosystem. Thus, we study ecosystem development based on historical series that represent the sequence of ecosystem states that actually occurred. Ecosystem states may be defined in various ways, including community composition, successional or developmental stages, and networks of biotic–abiotic interactions. Examples include ecological succession, ecological and soil chronosequences, biostratigraphy, paleoenvironmental reconstructions, and environmental histories. These historical sequences contain information—not just about the origin and development of the observed sequence but also about the state transitions embedded in the sequence and the general nature of ecosystem evolution. The purpose of this study is to develop and apply a method for measuring the complexity—directly related to entropy—of historical sequences and the expansion of state space, information, and complexity as evolution proceeds. The following two key questions are addressed: What are the trends in information and complexity as historical sequences are lengthened by higher-resolution, additional observations and the passage of time? For historical sequences with recurring or redundant states, what are the complexity and informational implications of rendering them as state-and-transition models (see Section 2.2)?

Ecosystem evolutionary sequences are often represented by a series of discrete states or stages based on, e.g., successional stages, biological community or land cover changes, habitat shifts, fossil assemblages, or paleosols. Although the processes are often continuous, the nature of historical records, palaeoecological archives, stratigraphic sequences, and observations or sampling often necessitate division into *N* discrete states. Each transition or sequence of transitions in the historical record provides some information and introduces some complexity. What is the relationship between the information/complexity of the subsequences embedded within the longer sequence as *N* increases?

Some developmental sequences contain redundant information. For example, transitions from state A to B may occur several times along the timeline. In such cases, it may be more appropriate to represent the evolutionary sequence as a state-and-transition graph. State transition graphs may be used to represent real or potential evolutionary sequences based on multiple observations or experiments, such as the methods often used to reconstruct speciation or vegetation community transition sequences [6,7]. State transition models (STMs) incorporate multiple causal pathways, which may or may not be sufficient to explain the entire pattern of the state transition graph. The methods presented here allow for the determination of whether the model is fully determined or under- or overdetermined with respect to the ability of the known pathways to explain the evolutionary sequence graph.

The questions tackled here can also help address the problematic issues identified by Beven [8] in the evolution of hydro-eco-geomorphological systems—that the contemporary state of environmental systems is historically contingent on events with varying degrees of persistence. A better understanding of the complexity of evolutionary sequences and the extent to which they can be explained based on causal pathways embedded within historical observations can help reduce the epistemic uncertainties [8]. Multiple definitions of complexity exist, with various degrees of overlap. In this paper, complexity is positively related to the number of elements in a system (or stages or states in a historical sequence), the number of interactions among components or states, and the potential of the system to produce nonlinear dynamics, randomness or pseudo-randomness, and emergence. Complexity is quantified here using the spectral radius of graphs and entropy-based measures.

The issue of complexities of evolutionary sequences parallels the rise of reduced-complexity models in ecology, hydrology, and geomorphology. Including more factors, processes, or constraints in a model tends to reduce the generality of the results, and simpler models often produce satisfactory or even more accurate results (e.g., [9,10,11,12]). However, the appropriate level of complexity (in terms of the number of variables, parameters, and processes included in the model) for the problem at hand is not always clear [12,13]. The analysis of embedded complexity within longer, higher-*N* evolutionary sequences allows assessment of the size vs. complexity tradeoffs involved and can also identify the appropriate level of complexity in underdetermined STMs or the possibilities for reducing redundancy in overdetermined STMs. Although they focus on the application of model-checking techniques from computer science, [14] highlights the insights gained from analyses of the structure of graph representations of ecological state transitions.

The methods and logic in this study are applicable to any historical sequence that can be represented as state changes or discrete stages, including evolutionary lineages. However, this work was motivated by an interest in interpreting environmental change and evolution at the ecosystem level. Thus, the examples and discussion will focus on ecosystems.

*Ecosystem* is used here in a broad sense to refer to environmental systems that include biological, abiotic, and hybrid components. Ecosystem *states* are qualitatively different from one another in terms of biotic community composition, soils, geomorphology, hydrology, or mass/energy flux patterns. *Outcome* is used to denote the observed state of an ecosystem at a specific time, usually contemporary or at the end of a particular evolutionary (sub-)sequence. The use of this word does not necessarily imply that the outcome state is an endpoint, as evolution is ongoing.

### Evolution and Information

Biological evolution has, in the aggregate, produced greater diversity and richness over time and more complex organisms. Biological evolution is based on the heritability of traits, and genes and DNA are often referred to in terms of coding and information transfer, as well as with metaphors such as “blueprints” or “instructions”. Thus, considerable research has addressed evolution from the perspective of information theory, including studies using information and algorithmic complexity as proxies for natural selection and biological information. This extends to ecology; Ulanowicz [15] suggested that “ecosystem behavior is the most palpable example of a purely natural ‘infodynamics’ that transcends classical dynamics but remains well within the realm of the quantifiable”.

Evolutionary research on broader scales (i.e., at the level of systems as opposed to individuals) has also increasingly been theorized in terms of information. In their study of structure, thermodynamics, and information in complex systems, Doménech et al. [16] maintained that “The basic principle of all evolution lies in the configuration of new structures with a greater degree of complexity and maximum effectiveness” for a given number of elements”. In each complex system, there is an associated generation complexity *E*(*S*), which is equal to the sum of the complexities within the complex system. They called this the complex system generating energy, as follows:

(1) $$E\left(\sum \right)=\sum_{\delta \in M}E\left(\delta \right)=\sum_{\delta =\left|M\right|}f_{\delta \;}E\left(\delta \right)\;$$

The number of different occurrences of element *δ* (frequency) in the complex system *S* is *f_δ_*. The sum of *f_δ_* is equal to |*M*|, the objects in the system. *E*(*δ*) is the complexity generated by element *δ* of set *M.* Doménech et al. [16] go on to propose a framework for interacting complex systems whereby information is transferred along gradients from the lower- to the higher-complexity system. *E*(*S*) is expected to increase as historical sequences become longer, as this cannot decrease embedded information. However, the extent to which information increases relative to total sequence length and complexity is not clear.

Similarly, although not arguing from thermodynamics, the “law of increasing functional information” was proposed by Wong et al. [17]. According to this proposed law, the functional information (negentropy) of a system will increase (i.e., the system will evolve) if many different configurations of the system undergo selection for one or more functions. This is based on the notion that at the system level, among multiple possible configurations, the one with the greatest functionality (utility in maintaining the system) will be selected.

Previous considerations of information and entropy, however, have focused on the information or complexity of individual stages of evolution or on the transfer of information from one stage or level to the next (e.g., [15,18]). This study, by contrast, considers the complexity and information of evolutionary and other historical sequences as a whole. The sequences are represented as graphs, and I use a common index of graph complexity, the spectral radius of the graph adjacency matrix (see below). The information content (and entropy) of a graph is closely related to the graph spectrum and the spectral radius. The close association of entropy, information, and complexity in this context should not be interpreted as suggesting the equivalence of these entities more generally, although they are often strongly related.

Classic debates in evolutionary biology and paleontology concern the extent to which change is incremental, slow, and gradual, versus episodes of large and rapid changes punctuating long periods of stasis, or various combinations thereof [19,20]. Similar debates exist in ecology and Earth sciences, although a key role for disturbances, catastrophic events, and other forms of episodic change is generally accepted [1,2,3,4,5,21,22]. However, even when change is gradual and continuous, historical records are almost always represented in serial terms. In some studies, this is an unavoidable outcome of the paleoenvironmental record, for example, in the form of stratigraphic sequences, annual layers in ice cores, or historical aerial photographs. In other cases, the serial nature of the record is tied to data collection, such as sampling at fixed depth intervals in peat profiles or ocean bed cores or repeat sampling of long-term research sites. In still other cases, the serial representation arises from the necessity or convenience of dividing or classifying subtle or complex phenomena into stages or categories. This is reflected in the identification of, e.g., successional sequences and ecological state-and-transition models.

## 2. Methods

Here we consider two types of representations of historical sequences, both in graph form. Serial data along a timeline are represented as simple, undirected linear sequential graphs. Each episode, sample, layer, or stage is a graph node, directly connected (only) to the immediately preceding and succeeding stage. These are treated as undirected graphs for greater generality. Some historical sequences are one-way and irreversible, but others are not. Further, representing the sequence as an undirected graph does not imply determinism or causality—it simply signifies that *B* follows *A*, not necessarily that *A* causes *B* or that *B* must follow *A*.

We also consider STMs, in which discrete system states (e.g., vegetation communities or soil types) and the transitions between them over time are identified. These are treated as directed graphs, as STMs reflect observed directions of changes among states and explicitly identify causes or triggers of transitions.

### 2.1. Linear Sequence

Let *S*(*t*) be an ecosystem stage or system state at time *t* of a historical sequence of length *N* such that *t* = 1, 2, …, *N*. *S*(*t*) is defined by qualitative changes, so that *S*(*t* − 1) ≠ *S*(*t*) ≠ *S*(*t* + 1), but otherwise there is no requirement or stipulation that a given *S* cannot recur. A vegetation sequence, for example, could repeat the same community, separated by other vegetation states or stages. Examples of linear sequences include seral stages in ecological succession, soil or ecological chronosequences, and paleosols or fossil assemblages in a stratigraphic sequence. The stages are not necessarily equal in length, nor are the differences between one stage or state and the next necessarily equal in magnitude. This depends on the purpose and methods used to develop the sequence.

A sequence *S*(*t*), *S*(*t* + 1), *S*(*t* + 2), …, *S*(*N*) can be represented as a linear sequential graph, with the states *S* as the nodes and the sequence of state changes as the links or edges connecting them.

The most versatile and robust measure of graph complexity is the spectral radius *λ*_1_, which is the largest eigenvalue of the graph adjacency matrix ***A***. A simple, unweighted graph ***A*** contains elements of 1 or 0 depending on whether the row and column elements are connected.

As discussed in Section 2.3, the spectral radius is directly related to entropy and information. *λ*_1_ is a common measure of graph complexity and is positively related to *N*, *m*, and the number of cycles in the graph.

Any connected graph *G* with *N* > 2 contains two or more subgraphs *G*′, defined as connected sets of nodes that are contained within *G.* Each of these represents information about transformations between two or more states. The sum of the spectral radii of the subgraphs (*Λ*) must be greater than or equal to the spectral radius of the parent graph, as follows:

*λ*_1_(*G*) ≤ *Λ* = *Σ λ*_1_(*G*′) (2)

For a linear sequential graph of overall length *N*, as follows:

*Λ*(*N*) = (*N* − 1) *λ*_1,2_ + (*N* − 1) *λ*_1,3_ + … + 2 *λ*_1,(*N*−1)_ (3)

where *λ*_1,*i*_ is the largest eigenvalue (spectral radius) of a linear sequential subgraph of length *i.* The embedded complexity index is the ratio *Λ*(*N*)/*λ*_1,*N*_, hereafter shown simply as *Λ*/*λ*_1_.

**Figure 1:**
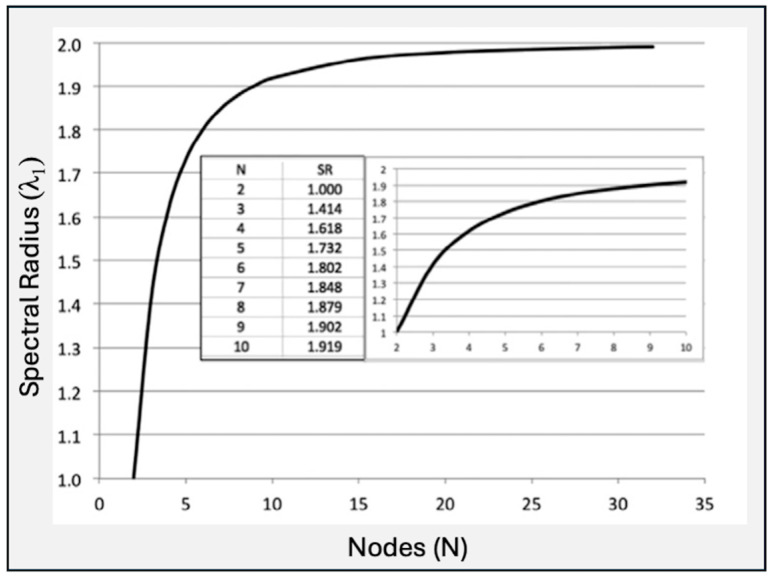
*Spectral radius of linear sequential graphs (reproduced with permission from* Phillips, J.D. 2016. Identifying sources of soil landscape complexity with spatial adjacency graphs. *Geoderma* 267, 58–64.) [23].

For linear sequential graphs, as shown in Figure 1:

(4) $$\underset{N\to \infty }{\lim}\lambda_{1\;}=2$$

For large *N*, the computation of *Λ* becomes cumbersome, and the embedded complexity index can be accurately estimated (*R*^2^ > 0.99) using a second-order polynomial, as follows:

*Λ*/*λ*_1_ = 1.4333 *N*^2^ − 4.1473 *N* + 2.059 (5)

### 2.2. State-and-Transition Models

Historical sequences can also be represented as state-and-transition models, with a linear sequential pattern being a special case of STM. STMs, a specific example of state transition graphs [14], were first developed by rangeland ecologists as an alternative to single-path succession models to describe more complex and variable patterns of change in vegetation communities. Overviews of STMs in ecology are given by [7,24,25]. An example is shown in the case study in Section 3.3. The authors of [26] explored the (often implicit) use of STMs, finding that they are generally veridical representations of empirical observations, rather than devices used to fit observations into predefined theoretical frameworks.

In the linear sequential case, each sub-sequence within the overall series represents information. Such historical sequences are often extended forward by the passage of time, backward by, e.g., deeper drilling or sampling or extension of chronologies, or in the middle by higher-resolution data collection or analytical techniques. The nature of STMs is that known pathways are embedded in the model. In rangeland STMs, for instance, there are often discrete pathways associated with increased or decreased fire frequency, grazing pressure, vegetation management, vegetation succession, and climate (e.g., drought). These are the relevant subgraphs for the directed graphs (digraphs) representing an STM.

Thus, for STMs, the equation is as follows:

(6) $$\frac{\Lambda }{\lambda_{1,STM\;}}=\frac{\left[\sum_{n=1}^{m}\lambda_{1,i}\right]}{\lambda_{1,STM}}$$

where *λ*_1,*i*_ are the spectral radii (largest eigenvalues) for the transition pathways (subgraphs) of the STM.

This is conceptually similar to the approach Phillips [23] developed to assess complexity in spatial adjacency graphs (SAG). In SAG, the graph nodes are elements such as vegetation communities, soil types, landforms, habitats, etc., which are connected if they occur contiguously in space. Embedded within these are gradients or factor sequences that explain some aspect of the spatial pattern. For example, these may be wet-to-dry sequences or elevation gradients of plant communities or soil types, or systematic variations in some key property, such as soil organic matter or salinity. These are essentially linear sequential subgraphs, such as transitions among rangeland states based on fire frequency or grazing pressure. Thus, in this case, Λ is the sum of the factor sequences, and the Λ/λ_1_ ratio shows whether the overall spatial adjacency pattern can be explained by the known factor sequences (if *Λ*/*λ*_1_ ≥ 1 this is the case). As SAGs are based only on contiguity, they are simple, undirected graphs. Ideally, an STM transition pattern is fully explicable based on causal pathways, so that *Λ* ≥ *λ*_1_.

An STM represented as a digraph with *N* nodes has *m* links, edges, or transitions as follows:

*N* − 1 ≤ *m* ≤ (*N*^2^ − *N*). (7)

The maximum spectral radius for a fully connected graph (each node can transition directly to all others) is *N* – 1, as follows:

0 ≤ *λ*_1,*N*(*STM*)_ ≤ *N* − 1 (8)

where *λ*_1,*LS*_(*N*), *λ*_1,*STM*_(*N*) are the spectral radii for a linear sequential graph of length *N* and for the STM.

Information (reduced complexity) compared to a fully connected network of the same *N* can be reduced by the presence of fewer than the maximum number of links *m* or by the specific pattern of connections (wiring). The relative importance of these can be determined using a method developed by [27].

The maximum spectral radius for an undirected graph with a given *N*, *m* is as follows:

(9) $$\lambda_{1,max}=\sqrt{\left\lceil 2m\frac{\left(N-1\right)}{N}\right\rceil }$$

which reduces to *N* − 1 for a fully connected (complete) graph. For a digraph:

(10) $$\lambda_{1\;}≾\;\sqrt{m},$$

or:

(11) $$\lambda_{1}\;\leq [{(d}_{max}^{-})(d_{max}^{+}){]}^{0.5}$$

where $d_{max}^{-},\;d_{max}^{+}$ indicate the maximum in and out degrees (number of incoming or outgoing links or edges) from any node [28].

If the spectral radius is less than the maximum, the relative contribution of the existence of fewer than the maximum number of edges and the specific network of connections (wiring) is given by the following equation:

(12) $$\zeta_{m}=\;\frac{\left[\left(N-1\right)-\;\lambda_{1,max}\right]}{\left(N-1\right)-\;\lambda_{1,STM}}$$

(13) $$\zeta_{wiring\;}=1-\;\zeta_{m}$$

*ζ_m_* and *ζ_wiring_* are the proportion of the reduction in STM spectral radius relative to the maximum accounted for by *m* less than the maximum possible and by the specific wiring of the connections, respectively. This is not an issue for linear sequential graphs, as the number of links and the wiring are a linear function of *N*.

### 2.3. Graph Energy, Entropy, and Information

Graph energy *E*(*G*) is the sum of the absolute values of all the real parts of the eigenvalues of a graph, as follows:

(14) $$E\left(G\right)=\;\sum_{i=1}^{N}{|\lambda }_{i\;}|$$

First devised as a measure of literal energy in physical chemistry, graph energy has since been applied in several fields, including as a measure of the total intensity of negative and positive feedbacks (reverberations) in ecosystems [21,29].

There exist many expressions for and concepts of graph entropy applicable to different types of graphs and applications [30,31,32]. The most straightforward, directly linked to the spectral radius, is the measure of entropy for digraphs [33], as follows:

*H*(*G*) = *log* *λ*_1_ (15)

Dehmer et al. [34] developed an entropy-based expression of graph structural information *Ig*(*G*), as follows:

(16) $$Ig\left(G\right)=\log(\sum_{i=1}^{N}\left|\lambda_{i}\right|)-\;\frac{1}{\sum_{i=1}^{N}\left|\lambda_{1}\right|}\;\sum_{i=1}^{N}\left|\lambda_{i}\right|\log\left|\lambda_{i}\right|,$$

which reduces to the following:

(17) $$Ig\left(G\right)=\log E\left(G\right)-\;\frac{1}{E\left(G\right)\;}\sum_{i=1}^{N}\left|\lambda_{i}\right|\log\left|\lambda_{i}\right|.$$

Dehmer and Mowshowitz [30,31] and Mowshowitz and Dehmer [32] identified other links between graph entropies and eigenvalues. Spectral radius is also closely related to singular value decomposition entropy of ecological networks [35].

## 3. Results: Case Studies

The methods above are applied to generic historical sequences and four empirical case studies, including two linear sequences and two STMs. These include a paleoecological study of community replacement in estuaries during sea-level transgression; succession in glacial forelands; woodland community shifts; and habitat changes in a delta. I also provide an example based on converting a linear sequence to an STM form.

### 3.1. Generic Historical Sequences

**Figure 2:**
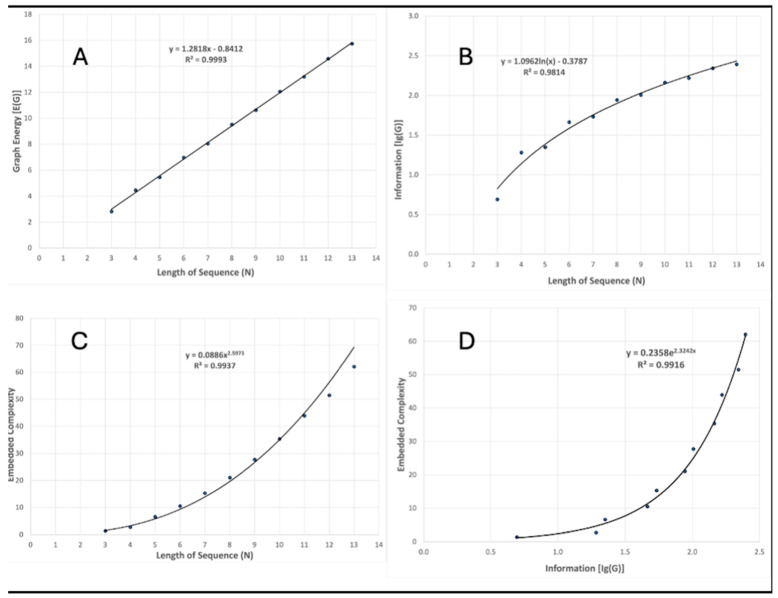
Trends in graph energy, information, and embedded complexity as sequence length increases (**A**–**C**); relationship between structural information and embedded complexity (**D**).

Spectral radius, graph energy, embedded complexity, and structural information (using Equation (17)) were computed for sequences of lengths 3 to 13 (the maximum appearing in the case studies in the next section). As sequence length (*N*) increases, λ_1_ asymptotically approaches two (see Figure 1), while the graph energy increases linearly (Figure 2A). Structural information increases with *N*, but at a slower rate than *E*(*G*). The best-fit line is a logarithmic function (Figure 2B).

Figure 2C shows the relationship between the embedded complexity index and sequence length. Λ/λ_1_ increases as approximately *N*^2.6^, indicating its sensitivity to sequence length. Figure 2D shows the strong relationship between information and embedded complexity. The shape of the curve and range of values show that the Λ/λ_1_ is a more sensitive indicator of the embedded information content.

### 3.2. Estuarine Community Replacement in a Transgressive Sequence

The coastal plain of North Carolina in the South Atlantic region of the USA has experienced multiple episodes of sea-level rise and fall in the Pleistocene. The Flanner Beach Formation is a geologic formation that provides a stratigraphic record of sea-level transgression. The formation is rich in invertebrate fossils, including bivalve mollusks, gastropods, and crustaceans. Miller [23,27] examined the fossil assemblages to develop a sequence of benthic community shifts associated with changes in salinity, circulation patterns, and substrate characteristics during an episode of sea-level rise beginning ca. 200 Ka.

Paleoecological sequences are often interpreted in terms of biological evolution or ecological succession, but Miller [36,37] emphasized community replacement. The latter involves the substitution of one community for another in gradually changing environments over sub-evolutionary time scales. Miller sampled fossil assemblages at 15 different stratigraphic levels, which represented nine benthic community stages, with community replacements driven by changes associated with sea-level rise and associated geomorphological and hydrographic changes at the coast. The communities are linked to environmental settings.

Underlying the Flanner Beach Formation is an older (early Pleistocene) transgressive sequence, the James City Formation. Although not as well studied as Flanner Beach, [27] identified three benthic community stages. A swamp paleosol is often found between the James City and Flanner Beach formations. Few faunal remains occur within it, but abundant in situ roots and stumps of bald cypress (*Taxodium distichum*) are preserved within the fine-grained organic-rich paleosol.

**Figure 3:**
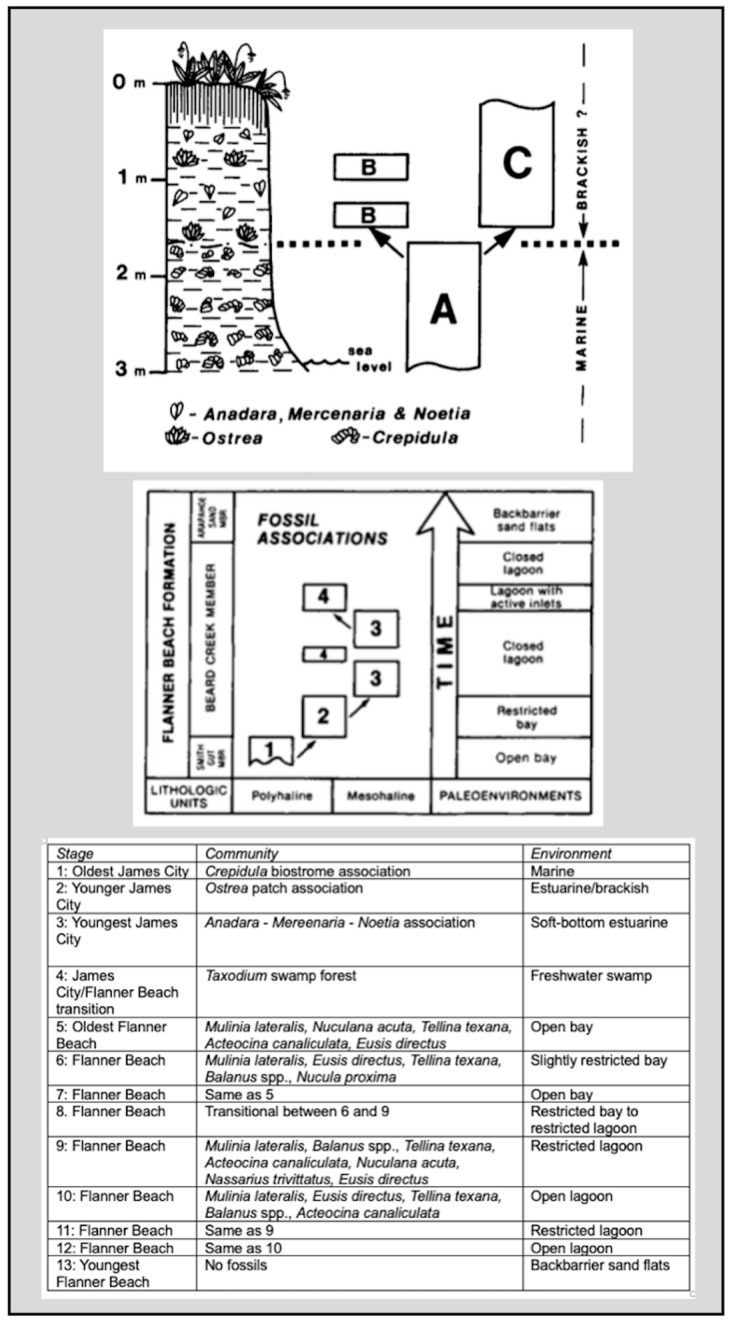
Community replacement sequence for Pleistocene benthic communities of the North Carolina coast, based on the work of William M. Miller III. Top and middle are simplified graphic depictions of stratigraphy from Miller [23,27] of the James City and Flanner Beach formations. At the bottom is the combined sequence of stages, with the swamp forest community inserted by the author. The Flanner Beach community names are based on the five most abundant fossil species.

The three James City stages and nine Flanner Beach stages, with the intervening swamp stage, are shown at the bottom of Figure 3.

For the James City sequence considered alone, *Λ*/*λ*_1_ = 2.5, and for the Flanner Beach Formation, *Λ*/*λ*_1_ = 80.8. Adding in the swamp forest stage for the whole *N* = 13 sequence, the embedded complexity index is 190.4. This illustrates the rapid nonlinear increase in information and complexity as the length of evolutionary sequences grows—either literally by the passage of time or virtually by the acquisition of additional observations or resolution. The index increases as *Λ*/*λ*_1_~*N*^2.6^. Thus, a threefold difference in state transitions from the James City to the Flanner Beach sequence yields a >32-fold increase in embedded complexity, but only a minuscule increase in overall complexity. Extending the Flanner Beach chronology by linking it to the James City with the swamp stage, a 44% increase in sequence length yields a 235% increase in embedded complexity.

This analysis does not address some issues inherent in paleoenvironmental studies, such as the lack of stratigraphic representation of erosional vs. depositional episodes, differential preservation of organic remains, etc. It does illustrate the disproportionate information gains associated with extending or refining evolutionary chronologies. For instance, the restricted lagoon community recurs in the Flanner Beach Formation, which provides no new information about benthic ecology but reveals the community changes associated with geomorphological changes in barrier islands, inlets, and estuarine conditions—important information in interpreting past and future responses to sea-level change. The Flanner Beach sequence is revisited in Section 3.6.

### 3.3. Glacial Forefield Chronosequences

Glaciers around the world are, for the most part, retreating due to global warming. As alpine glaciers retreat, areas once covered by ice are newly re-exposed, with colonization by pioneer species and subsequent ecological succession, along with associated soil, hydrological, and geomorphic processes. The exposed ground represents a chronosequence, as the areas near the glacier are younger in terms of subaerial exposure, with increasingly longer exposure ages with distance. These situations make excellent field sites for studies of the early stages of ecosystem evolution.

**Figure 4:**
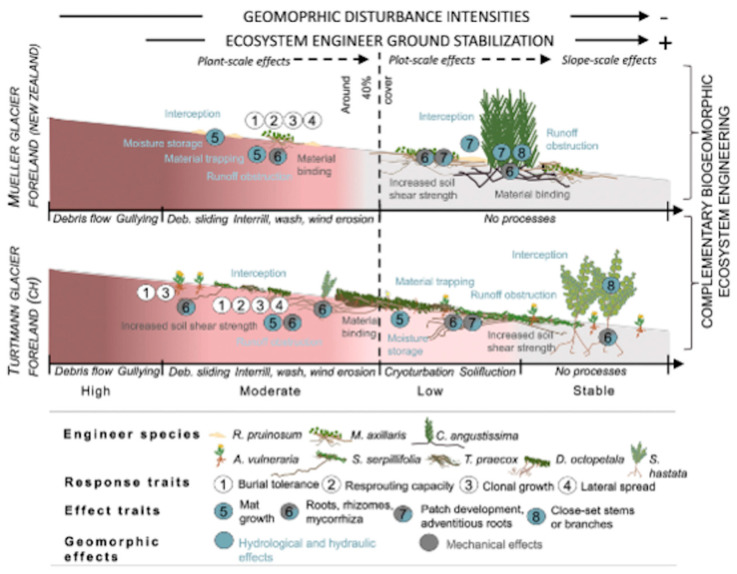
Two examples of glacial forefield succession, emphasizing the interaction of biological processes and geomorphic disturbances (Reproduced under a CC BY 4.0 license from Eichel, J.; Draebing, D.; Winkler, S.; Meyer, N. 2023. Similar vegetation–geomorphic disturbance feedbacks shape unstable glacier forelands across mountain regions. Ecosphere 14, e4404.) [39].

In their review of factors affecting succession in glacial forefields, [38] identified four basic stages (in the absence of disturbances)—pioneer, intermediate, advanced, and mature. This is a generalized *N* = 4 sequence, although (as the authors acknowledge) the details differ among sites and further discrimination within the four basic stages is possible. Note also that advanced and mature should be understood in relative terms, as most of the studied chronosequences are <200 years long. Jana Eichel and others [39,40] emphasized the role of geomorphological disturbances in delaying or altering successional trajectories (Figure 4), but their general conceptual models all resolve to *N* = 4 examples.

Eichel [40] identified five stages (pioneer, early successional, two intermediate successional, and late successional) based on systematic temporal changes in the relative importance of abiotic environmental controls, biotic factors, and biotic/abiotic interactions (again, acknowledging geographical and within-stage variations). These variations and biogeomorphological feedbacks are explored in greater detail in [39].

One example of a site-specific study is that of Matthews and Vater [41] in southern Norway, which illustrates how more detailed evolutionary sequences can be explicated. They were able to identify eight distinct terrain age zones in a 33-year chronosequence. In general successional models, all eight chronosequence members (aged 1 to 33 years) would be classified as pioneer or early successional stages. However, their data (40: Table 1) show distinct changes in presence/absence and relative abundance of higher plants, lower plants, and invertebrates from each member to the next, such that they represent an *N* = 8 sequence of community development.

Table 1 shows results for the chronosequences above, as well as for a hypothetical *N* = 40 chronosequence, which could be envisioned for, say, a 40-year sequence sampled annually, or a 200-year time span with a mean sample interval of five years. This further illustrates the disproportionate increase in embedded complexity in longer evolutionary sequences—for instance, a fivefold increase from *N* = 8 to 40 results in a slight increase in spectral radius and a <2× increase in structural information, while the embedded complexity index increases >25-fold.

### 3.4. Longleaf Pine Woodland State Transitions

**Figure 5:**
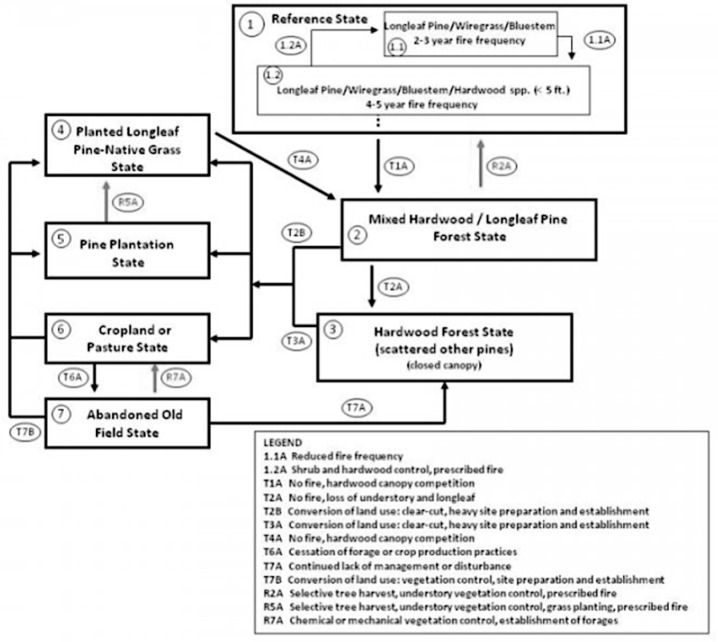
State-and-transition model for low rise, moderately wet ecological site in the Atlantic Coast Flatwoods Major Land Resource Area. Reproduced from the ecological site description (https://edit.jornada.nmsu.edu/catalogs/esd/153A/R153AY001GA; accessed on 24 March 2024).

Figure 5 shows an STM for longleaf pine woodlands in the southeastern U.S.A. These systems are found in South Carolina and Georgia in U.S. Department of Agriculture Major Land Resource Area 153A—Atlantic Coast Flatwoods. The STM applies to the ecological site designation loamy rise, moderately wet, described in detail in the ecological site description from the Ecosystem Dynamics Interpretation Tool (https://edit.jornada.nmsu.edu/catalogs/esd/153A/R153AY001GA; accessed on 24 March 2024).

The STM shown in Figure 5 has *N* = 8 with an interaction matrix shown in Table 2. The spectral radius of this graph is 2.321, and *E*(*G*) = 6.848.

For STMs, the relevant subgraphs (evolutionary pathways based on observed transitions) can be discerned from the data and information used to derive the model, in this example, from the ecological site description. These are shown in the legend of Figure 5 and as subgraph pathways in Table 3.

*Λ* = 28.516, and the embedded complexity index is 12.3. The index for this STM is less than that for a linear sequence of the same number of nodes, suggesting another useful index, (*Λ*/*λ*_1_)*_STM_*/(*Λ*/*λ*_1_)*_LS_*. In this case, that index is 12.286/21.077 = 0.583. The graph representing the STM looks more complex than a linear historical sequence with the same number of nodes, but the embedded complexity is less, indicating a degree of redundancy in the transition pattern.

The STM of Figure 5 has ζ_m_ = 0.540, and ζ_wiring_ = 0.460. This indicates that 54 percent of the reduction in spectral radius from the maximum for an all-transitions-are-possible case is due to having fewer than the maximum number of links, and 46 percent is attributable to the specific wiring.

### 3.5. San Antonio River Delta

This case study is based on research on habitat changes in the San Antonio River Delta, Texas. Ecosystem transitions in this area are driven by rising sea levels, changes in riverine flow inputs (strongly influenced by climate, land use, and upstream water allocations), tectonic influences, the effects of large woody debris, and fluvial geomorphological dynamics, including deposition, erosion, and avulsions (channel changes). Background information is given in [22,42,43,44].

**Figure 6:**
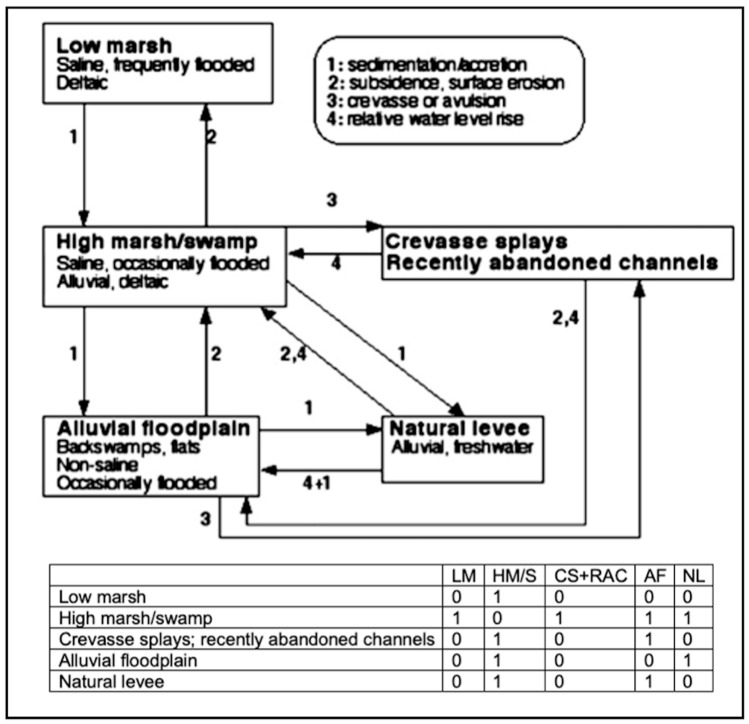
State transition model for the San Antonio River Delta (top), after 33: Figure 2. Bottom shows the adjacency matrix.

An STM for this system shows five distinct habitats (with associated ecological communities, landforms, soils, and hydrological regimes) in the delta area, with observed transitions among them, linked to hydrogeomorphic processes (Figure 6). Table 4 shows the specific subsequences included in the STM.

For the delta STM, λ_1_ = 2.286, *Λ* = 24.720, Λ/λ_1_ = 10.814, and (*Λ*/*λ*_1_)*_STM_*/(*Λ*/*λ*_1_)*_LS_* = 1.632. In contrast to the preceding example, the STM has higher embedded complexity than a linear sequence of the same *N*. However, in this case, more of the reduction of the spectral radius from the maximum possible is attributable to the graph wiring than to a reduction of *m* from the maximum (ζ_m_ = 0.399; ζ_wiring_ = 0.601).

### 3.6. Flanner Beach Revisited

**Figure 7:**
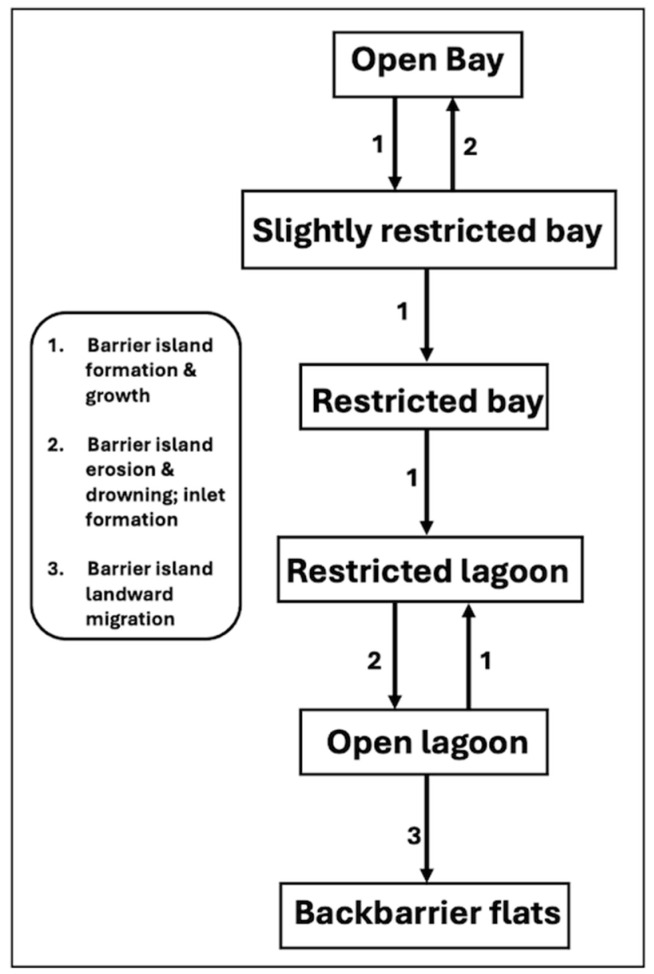
Historical sequence of benthic communities of the Flanner Beach Formation (see Figure 3), interpreted as a state-and-transition model.

In this section, we take the linear sequence of community replacement in the Flanner Beach Formation from Section 4.2, which has several recurrences of the same community, and recast it as an STM, as shown in Figure 7. The environmental changes associated with the community replacements can be interpreted in terms of hydrographic changes associated with the growth, migration, erosion, and drowning of barrier islands and the opening and closure of barrier island inlets [45].

The STM shown in Figure 7 has a spectral radius of one, graph energy of four, and structural information of two. The embedded complexity index, based on the transition drivers of tidal and ocean exchange restriction by barrier island formation and expansion, increased by barrier island erosion, drowning, and inlet formation, and landward migration of barriers, is 5.168.

**Figure 8:**
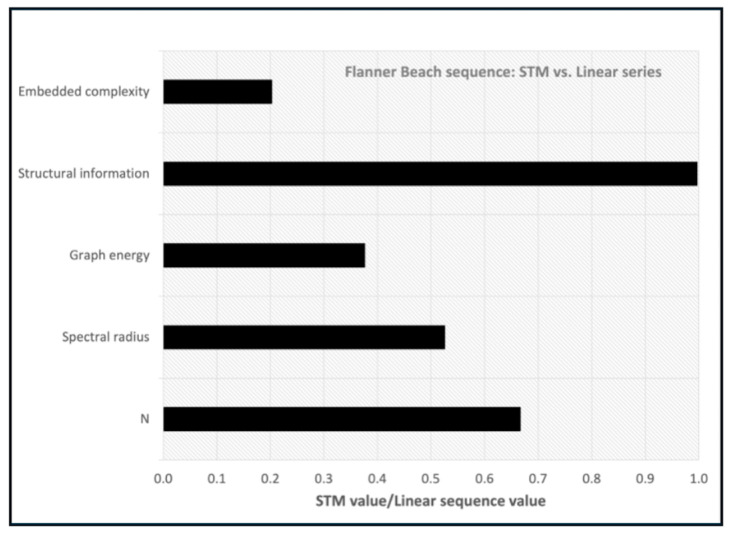
Comparison between Flanner Beach community replacement sequence represented as a linear series and state-and-transition model.

Comparing the two different representations of the Flanner Beach community transitions (undirected linear sequence vs. STM), the STM version collapses *N* from nine to six and produces a substantially lower spectral radius, graph energy, and embedded complexity index (Figure 8). The structural information *Ig*(*G*) is nearly equal.

## 4. Discussion

### 4.1. (When) Is More Better?

Scientists long operated under the assumption that more is better—more data, longer time series, more complete models, etc. This attitude still dominates, but not as strongly as it once did. As more information accumulates and more options or variables are identified and considered, and as increasingly higher resolutions and denser data matrices accumulate, there are inefficiencies due to redundancy, limits to information storage and processing, and the well-known tradeoffs between data quantity and model complexity on one hand versus computing time on the other.

More fundamentally, denser or higher-resolution data do not always produce more accurate, insightful, or useful results. With respect to digital elevation models, for example, the highest available resolutions are not always optimal for ecosystem modeling or terrain representation (e.g., [46,47,48]). Several studies have also found that, in addition to advantages in computational efficiencies and implementation costs, reduced-complexity models provide more accurate and useful results in many environmental modeling contexts (e.g., [9,10,11,12,49,50]).

For ecosystems and other Earth surface systems, Phillips (28: ch. 9) pointed out that any given system is a function of the combined effects of *i* = 1, 2, …, *n* applicable global or general laws *X_g_*, *j* = 1, 2, …, *m* geographically contingent environmental (place) factors *X_p_*, and *k* = 1, 2, …, *q* historical events or contingencies *X_h_*. The probability (*p*) of occurrence or existence of a specific ecosystem state is, then, the following:

(18) $$p(S)=\;\prod_{i=1}^{n}p(X_{g})\;\prod_{j=1}^{m}p\left(X_{p}\right)\prod_{k=1}^{q}p(X_{h})$$

The probability of any particular combination of geographically contingent place factors and path-dependent historical factors recurring in another location becomes vanishingly small as more factors or variables are included. This reasoning even pertains indirectly to the law factors, as they must be applied in specific environmental contexts. The more details are added the more improbable or unique the ecosystem representation becomes, at the expense of general applicability.

Thus, clearly, more is not always better. How does this apply to evolutionary sequences?

For linear sequences, the embedded complexity increases rapidly with longer or more detailed series (higher-*N*). For example, in the proglacial succession example, a fivefold increase from *N* = 8 to 40 results in a slight increase in spectral radius and a <2× increase in structural information, while the embedded complexity index increases >25-fold. This shows that in similar cases more *is* better in some respects. As the overall complexity of the sequence increases only slightly with more than a few nodes, the longer sequences are more efficient containers and conveyors of information, in that the information increase has a negligible cost in increased complexity. However, due to redundant transitions in some sequences, representation as STMs might provide advantages, whereupon more might not be better.

### 4.2. STM Advantages

The box-and-arrow models representing STMs look more complex than linear sequences and were initially developed to depict ecological changes as richer and more varied than single-path, linear succession trends. All but the simplest STMs (linear sequences and simple radiation or convergence patterns can be considered special cases of STMs) indeed have greater spectral radius and entropy values, indicating greater complexity. STMs also have higher graph energy than linear sequences of commensurate *N*, reflecting greater reverberation.

Recalling that structural information is closely related to graph energy, STMs also have higher *Ig*(*G*) than linear sequences. The embedded complexity of STMs may be higher or lower than that of a linear sequence of the same *N*. The comparison of the results of the longleaf pine and river delta STMs here suggests that this may depend on how many subgraph transition sequences are associated with the transition pathways within the STM. An underdetermined STM (*Λ*/*λ*_1,*STM*_ < 1) indicates the need to explore further causal explanations for the transitions depicted.

Evolutionary sequences in which certain states recur may allow for the sequence to be collapsed into an STM. This is a mechanism to reduce the *N* of an evolutionary graph, although only a radiation-type pattern (all states either derive from or converge to a single key state), is comparably simple to a linear sequence. Representing the Flanner Beach sequence as a state transition graph reduces *N* from nine to six and yields a substantially lower spectral radius. The structural information *Ig*(*G*) is nearly equal. The STM form would thus be advantageous for some purposes, such as predicting changes in benthic communities resulting from the geomorphic evolution of the coast. However, the linear sequential form would be better suited to match the chronology of benthic ecological changes with other chronologies based on, e.g., lithostratigraphy or foraminifera.

The other two STMs examined here provide contrasting cases. The longleaf pine woodland and the San Antonio River Delta STMs have greater complexity than linear sequential graphs of the same *N*. In the former case, however, its embedded complexity is lower, while in the delta example, the embedded complexity is higher. The ζ indices can reveal the extent to which spectral radius and entropy less than the maximum possible is due to having fewer than the maximum connections versus the wiring of the connections. This can provide guidance for further studies. For example, the higher *ζ_wiring_* highlights the fact that the same processes (e.g., sedimentation) can result in transitions along multiple pathways.

The STM for the longleaf pine woodland has a higher spectral radius, graph energy, and structural information content than a linear sequence of the same number of states (*N* = 8). However, the embedded complexity index is lower, reflecting greater efficiency and less redundancy. The *Λ* value for a linear sequence includes the subgraphs representing many overlapping transitions, so representation as an STM consolidates some of that information and reduces the embedded complexity.

## 5. Conclusions

Although multiple evolutionary pathways are possible, for any observed historical sequence, only one occurred. A measure of the embedded complexity of historical sequences is based on the overall complexity of the series (represented as a graph, with complexity measured by the spectral radius) relative to the combined complexity of the subgraphs within it. The embedded complexity index is closely linked to graph entropy, so the index also reflects information in the sequence. The structural information of the sequence is positively related to the Λ/λ_1_ index, but the latter is more numerically sensitive and may provide greater discrimination. As historical sequences are lengthened, the overall complexity asymptotically approaches *λ*_1_ = 2, and the graph energy increases linearly. Embedded complexity, by contrast, increases as *N*^2.6^.

Embedded complexity can also be computed for ecological state-and-transition models, representing observed transitions, along with information on their causes or triggers. Depending on their specific pattern of transitions and the causal subgraphs embedded within them, they may have greater or less embedded complexity than a linear sequence of the same number of nodes. However, if enough is known about the triggers or causes of the observed state transitions, linear sequences with recurring states may be recast as STMs for a more efficient representation.

## Figures and Tables

**Table 1:** Embedded complexity and related measures for glacial forefield chronosequences of variable lengths.

*Chronosequence*	*N*	*λ*_1_	*E*(*G*)	*Ig*(*G*)	*Λ*	*Λ*/*λ*_1_
4-stage general succession model	4	1.618	3.236	1.283	4.414	2.728
5-stage general succession model	5	1.732	3.464	1.350	11.478	6.627
Matthews and Vater, 2015	8	1.879	3.758	1.944	39.604	21.077
Hypothetical	40	~2	3.998	3.650	1064	532.666

**Table 2:** Adjacency matrix for the state transition model shown in Figure 5. Entries are 1 if the column state can transition to the row state and 0 otherwise.

	1.1	1.2	2	3	4	5	6	7
1.1 Longleaf pine/wiregrass/bluestem 2–3 yr fire frequency	0	1	0	0	0	0	0	0
1.2 Longleaf pine/wiregrass/bluestem/ sardwood spp.; 4–5 yr fire frequency	1	0	1	0	0	0	0	0
2 Mixed hardwood/longleaf pine forest	1	1	0	1	1	1	1	0
3 Hardwood forest	0	0	0	0	1	1	1	0
4 Planted longleaf pine-native grass	0	0	1	0	0	0	0	0
5 Pine plantation	0	0	0	0	1	0	0	0
6 Cropland or pasture	0	0	0	0	1	1	0	1
7 Abandoned old field	0	0	0	1	0	1	1	0

**Table 3:** State transition pathways within the longleaf pine woodland state transition model. Numbers refer to the states shown in Figure 5 and Table 3.

*Pathway (Sub-Sequence) Types*	*N* = 2	*N* = 3	*N* = 4
Fire suppression; reduced fire frequency	1.1→1.2 4→2	1.1→1.2→2 4→2→3	1.1→1.2→2→3
Prescribed fire; increased fire frequency	1.2→1.1 2→1.1 2→1.2	2→1.2→1.1	None
Silvicultural activities	2→4 2→5 3→5 6→5 6→4 7→5 7→6	2→5→4 3→5→4 6→5→4	None
Agricultural activities	2→5 3→5 7→5	None	None
Non-disturbance, succession	7→3 4→2	4→2→3	None
*Number of linear subgraphs*	17	7	1
*Sum of spectral radii*	17	9.898	1.618

**Table 4:** State transition pathways within San Antonio River Delta state transition model. Acronyms refer to the states shown in Figure 4.

*Pathway (Sub-Sequence) Types*	*N* = 2	*N* = 3	*N* = 4
Deposition, sedimentation, accretion	LM→HM/SW HM/SW→AF HM/SW→NL AF→NL	LM→HH/SW→NL LM→HH/SW→AF HM/SW→AF→NL	LM→HM/SW→AF→NL
Erosion, subsidence	AF→HM/SW NL→HM/SW CS/RAC→AF	AF→HM/SW→LM NL→HM/SW→LM CS/RAF→AF→ HM/SW	CS/RAF→AF→ HM/SW→LM
Crevasse-avulsion	HM/SW→ CS/RAC AF→CS/RAC	None	None
Relative water level rise	CS/RAC→ HM/SW CS/RAC→AF NL→HM/SW NL→AF	None	None
*Number of linear subgraphs*	13	6	2
*Sum of spectral radii*	13	8.484	3.236

## Data Availability

All data created for this research are presented in the article.

## References

[1] Mori A.S. (2011). Ecosystem management based on natural disturbances: Hierarchical context and non-equilibrium paradigm. J. Appl. Ecol..

[2] Šamonil P., Doleželová P., Vašíčková I., Adam D., Valtera M., Král K., Janík D., Šebková B. (2013). Individual-based approach to the detection of disturbance history through spatial scales in a natural beech-dominated forest. J. Veg. Sci..

[3] Pulsford S.A., Lindenmayer D.B., Driscoll D.A. (2016). A succession of theories: Purging redundancy from disturbance theory. Biol. Rev..

[4] Estrada-Villegas S., Bailón M., Hall J.S., Schnitzer S.A., Turner B.L., Caughlin T., van Breugel M. (2020). Edaphic factors and initial conditions influence successional trajectories of early regenerating tropical dry forests. J. Ecol..

[5] Sánchez-Pinillos M., Dakos V., Kéfi S.K. (2024). Ecological dynamic regimes: A key concept for assessing ecological resilience. Biol. Conserv..

[6] Stankowski S., Ravinet M. (2021). The speciation continuum. Evolution.

[7] Briske D.D., Fulendor S.D., Smeins F.E. (2005). State-and-transition models, thresholds, and rangeland health: A synthesis of ecological concepts and perspectives. Rangel. Ecol. Manag..

[8] Beven K.J. (2015). What we see now: Event-persistence and the predictability of hydro-eco-geomorphological systems. Ecol. Mod..

[9] Hong E.-M., Pachespsky Y.A., Whelan G., Nicholson T. (2017). Simpler models in environmental studies and prediction. Crit. Rev. Environ. Sci. Technol..

[10] McClure R., Naylor D., Farris Y., Davison M., Fansler S.J., Hofmockel K.S., Jansson J.K. (2020). Development and analysis of a stable, reduced-complexity model soil microbiome. Front. Microbiol..

[11] Goodrum G.C., Null S.E. (2023). Reduced complexity models for regional aquatic habitat selection. J. Am. Water Resour. Assoc..

[12] Sivakumar B. (2004). Dominant processes concept in hydrology: Moving forward. Hydrol. Proc..

[13] French J., Payo A., Murray B., Orford J., Eliot M., Cowell P. (2015). Appropriate complexity for the prediction of coastal and estuarine geomorphic behaviour at decadal to centennial scales. Geomorphology.

[14] Thomas C., Cosme M., Gaucherel C., Pommereau F. (2022). Model-checking ecological state-transition graphs. PLoS Comput. Biol..

[15] Ulanowicz R.E., Dehmer M., Emmert-Streib F., Mehler A. (2011). The central role of information theory in ecology. Towards an Information Theory of Complex Networks: Statistical Methods and Applications.

[16] Doménech J.L.U., Nescolarde-Selva J.A., Lloret-Climent M. (2022). Structure, thermodynamics and information in complex systems. Kybernetes.

[17] Wong M.L., Cleland C.E., Arend D., Bartlett S., Cleaves H.J., Demarest H., Prabhu A., Lunine J.I., Hazen R.M. (2023). On the roles of function and selection in evolving systems. Proc. Nat. Acad. Sci. USA.

[18] Lineweaver C.H., Davies P.C.W., Ruse M. (2013). Complexity and the Arrow of Time.

[19] Huggett R.J. (1990). Catastrophism. Systems of Earth History.

[20] Palmer T. (1999). Controversy. Catastrophism and Evolution.

[21] Phillips J.D. (2021). Landscape Evolution. Landforms, Ecosystems, Soils.

[22] Phillips J.D. (2014). State transitions in geomorphic responses to environmental change. Geomorphology.

[23] Phillips J.D. (2016). Identifying sources of soil landscape complexity with spatial adjacency graphs. Geoderma.

[24] Bestelmeyer B.T., Tugel A.J., Peacock D.G., Robinett D.G., Shaver P.L., Brown J.R., Herrick J.E., Sanchez H., Havstad K.M. (2009). State-and-transition models for heterogeneous landscapes: A strategy for development and application. Rangel. Ecol. Manag..

[25] Bestelmeyer B., Fernández-Giménez M., Densambuu B., Bruegger R. (2021). State-and-transition modelling. The Routledge Handbook of Research Methods for Social-Ecological Systems.

[26] Phillips J.D., Van Dyke C. (2017). Geomorphological state-and-transition models. Catena.

[27] Phillips J.D. (2012). Synchronization and scale in geomorphic systems. Geomorphology.

[28] Kwapisz J. (1996). On the spectral radius of a directed graph. J. Graph Theor..

[29] Phillips J.D. (2019). Evolutionary pathways in soil-geomorphic systems. Soil Sci..

[30] Dehmer M., Mowshowitz A. (2011). A history of graph entropy measures. Inf. Sci..

[31] Dehmer M., Mowshowitz A. (2011). Generalized graph entropies. Complexity.

[32] Mowshowitz A., Dehmer M. (2012). Entropy and the complexity of graphs revisited. Entropy.

[33] Geller W., Kitchens B., Misiurewicz M., Rams M. (2012). A spectral radius estimate and entropy of hypercubes. Int. J. Bifurc. Chaos.

[34] Dehmer M., Emmert-Streib F., Shi Y. (2014). Interrelations of graph distance measures based on topological indices. PLoS ONE.

[35] Strydom T., Dalla Riva G.V., Poisot T. (2021). SVD entropy reveals the high complexity of ecological networks. Front. Ecol. Evol..

[36] Miller W.M. (1986). Community replacement in estuarine Pleistocene deposits of eastern North Carolina. Tulane Stud. Geol. Paleontol..

[37] Miller W.M. (1986). Paleoecology of benthic community replacement. Lethaia.

[38] Wojcik R., Eichel J., Bradley J.A., Benning L.G. (2021). How allogenic factors affect succession in glacier forefields. Earth-Sci. Rev..

[39] Eichel J., Draebing D., Winkler S., Meyer N. (2023). Similar vegetation-geomorphic disturbance feedbacks shape unstable glacier forelands across mountain regions. Ecosphere.

[40] Eichel J., Heckmann T., Morche D. (2019). Vegetation succession and biogeomorphic interactions in glacial forelands. Geomorphology of Proglacial Systems.

[41] Matthews J.A., Vater A.E. (2015). Pioneer zone geo-ecological change: Observations from a chronosequence on the Storbreen glacial foreland, Jutunheimen, southern Norway. Catena.

[42] Phillips J.D. (2011). The structure of ecological state transitions: Amplification, synchronization, and constraints. Ecol. Comp..

[43] Phillips J.D. (2011). Predicting modes of spatial change from state-and-transition models. Ecol. Mod..

[44] Phillips J.D. (2012). Logjams and avulsions in the San Antonio River delta. Earth Surf. Proc. Landf..

[45] Zaremba N., Mallinson D.J., Leorri E., Culver S., Riggs S., Mulligan R., Horsman E., Mitra S. (2016). Controls on the stratigraphic framework and paleoenvironmental change within a Holocene estuarine system: Pamlico Sound, North Carolina, USA. Mar. Geol..

[46] Kim D., Zheng Y.B. (2011). Scale-dependent predictability of DEM-based landform attributes for soil spatial variability in a coastal dune system. Geoderma.

[47] Möller M., Volk M. (2015). Effective map scales for soil transport processes and related process domains—Statistical and spatial characterization of their scale-specific inaccuracies. Geoderma.

[48] Wu Y., Zhou L., Meng Y., Lin Q., Fei Y. (2023). Influential topographic factor identification of soil heavy metals using GeoDetector: The effects of DEM resolution and pollution sources. Remote Sens..

[49] Bokhari H.H., Najafi E., Dawidowicz J., Wuchen L., Maxfield N., Vörösmarty C.J., Fekete B.M., Corsi F., Sanyal S., Lin T.-S. (2023). Simulating basin-scale linkages of the food-energy-water nexus with reduced complexity modeling. Front. Environ. Sci..

[50] Nicholas A.P., Sandbach S.D., Ashworth P.J., Amsler M.L., Best J.L., Hardy R.J., Lane S.N., Orfeo O., Parsons D.R., Reesink A.J. (2012). Modelling hydrodynamics in the Rio Parana, Argentina: An evaluation and inter-comparison of reduced-complexity and physics based models applied to a large sand-bed river. Geomorphology.

